# A Space-For-Time (SFT) Substitution Approach to Studying Historical Phenological Changes in Urban Environment

**DOI:** 10.1371/journal.pone.0051260

**Published:** 2012-12-07

**Authors:** Alexander Buyantuyev, Pengyan Xu, Jianguo Wu, Shunji Piao, Dachuan Wang

**Affiliations:** 1 Sino-US Center for Conservation, Energy, and Sustainability Science, Inner Mongolia University, Hohhot, China; 2 School of Life Sciences, Inner Mongolia University, Hohhot, China; 3 School of Life Sciences and Global Institute of Sustainability, Arizona State University, Tempe, Arizona, United States of America; Lakehead University, Canada

## Abstract

Plant phenological records are crucial for predicting plant responses to global warming. However, many historical records are either short or replete with data gaps, which pose limitations and may lead to erroneous conclusions about the direction and magnitude of change. In addition to uninterrupted monitoring, missing observations may be substituted via modeling, experimentation, or gradient analysis. Here we have developed a space-for-time (SFT) substitution method that uses spatial phenology and temperature data to fill gaps in historical records. To do this, we combined historical data for several tree species from a single location with spatial data for the same species and used linear regression and Analysis of Covariance (ANCOVA) to build complementary spring phenology models and assess improvements achieved by the approach. SFT substitution allowed increasing the sample size and developing more robust phenology models for some of the species studied. Testing models with reduced historical data size revealed thresholds at which SFT improved historical trend estimation. We conclude that under certain circumstances both the robustness of models and accuracy of phenological trends can be enhanced although some limitations and assumptions still need to be resolved. There is considerable potential for exploring SFT analyses in phenology studies, especially those conducted in urban environments and those dealing with non-linearities in phenology modeling.

## Introduction

Historical trends in plant phenology (environment-mediated chronology of periodic life-history events (phenophases)) have become widely used for assessing climate change and its effects on ecological processes [1]–[4]. Such analyses rely on long-term records of phenological observations accumulated within national networks or collected by individual researchers [5]–[8]. Global warming most often advances spring (earlier budbreak and earlier emergence of leaves and flowers) and slightly delays autumn phenophases (later leaf coloration and leaf drop) with the overall effect of growing season lengthening in mid- to higher latitudes [9]–[11]. Yet, in some areas warmer cold period conditions may prevent the fulfillment of chilling requirements and, as a consequence, delay spring phenophases [12].

Historical records often span relatively short periods or have missing data preventing researchers from making meaningful statistical inferences when they regress phenophase timing on years [13]. Consequently, it may be problematic to evaluate historical trends in phenology and extrapolate them to the future because some records may miss the full magnitude of climatic cycles or extreme events. This raises concerns about the validity of some published literature with conclusions based on less than 10 years of data [14].

A typical approach to overcome the problem of scarce phenological time-series has been to combine data from multiple stations and either derive a mean regional signal [9] or use alternative methods of time-series optimization, which also allows reducing the noise and removing outliers in data [15]–[17]. Recently, records other than direct phenology observations, including dated historical photographs and herbarium specimens, have been used to estimate historical changes in phenology [18], [19]. However, the temporal accuracy of such data is generally significantly lower. The exclusive reliance on statistical techniques, that do not require combining data from multiple stations, is also an option for estimating missing values. They include imputation-based procedures, weighting procedures, and model-based procedures. In the imputation-based approach missing values are filled in using any of the imputation techniques (hot-deck, mean, and regression), followed by data analysis using standard methods [20]. The more advanced approach is model-based procedures, such as the expectation-maximization (EM) maximum likelihood method [21]. However, when too many important observations are missing, obtaining such data can only be done through controlled field experiments, mainly artificial warming of natural plant populations or communities [2], [22], [23], or by making use of naturally occurring latitudinal/altitudinal gradients of temperature [5], [24]. A recent meta-analysis of warming experimentation in phenology research revealed its lower sensitivity compared to long-term observations [25]. Some of the limitations of experimentation and gradient analysis can be alleviated by integrating them in a single study [26]. Experimental approach is feasible in herbaceous and shrub communities, but it is much more difficult to apply in forest stands. Furthermore, it is challenging to experiment with effects of cooling, which may be of interest if data for historical lows are not available. On the other hand, the use of spatial climatic gradients may be a more achievable option in evaluating tree responses to changes in climate.

It has been suggested that elevated temperatures and CO_2_ concentrations in urban areas provide a low-cost alternative to experimental studies of climate change effects [27]. From a landscape ecology perspective, the urban-rural temperature gradient, known as the Urban Heat Island (UHI), may be considered as a broad-scale experiment that provides an opportunity not only for assessing the effects of global warming but also for filling gaps in phenological records. While phenological observations have been traditionally conducted in the vicinity of human settlements, most such data are spatially isolated point features. Several recent studies focused specifically on spatial variation of temperature and phenology across urban areas and reported that variations in spring phenology were correlated with temperature patterns [27]–[29]. Most such investigations support the prevailing view that urban warming leads to earlier onset of spring phenophases and longer growing season, with a few exceptions [30].

We propose that scarce historical records can be combined with data collected during one or more growing seasons over many locations along urban-rural temperature gradients. Phenological models developed using this combined phenology and climate data can help us restore missing observations of the past and thus estimate phenology trends more accurately. This should be straightforward if monitored plants commonly grow in a metropolitan area as either managed or remnant urban vegetation. Otherwise, experimental approach [27], [29] may be an option. Use of spatial data for inferring temporal dynamics has a long tradition in ecology. The approach is commonly known as the space-for-time (SFT) substitution whereby spatially separated sites selected based on either ecological or environmental gradients serve as proxies for predicting ecological time-series, e.g. vegetation succession [31], [32]. Therefore, the primary goal of our study is to test the usefulness of the SFT in studying urban phenology. Specifically, we combine historical temperature and spring phenological records of several tree species with temperature data and phenological observations of same species conducted during two growing seasons at multiple sites along an urban-rural temperature gradient. We use these combined datasets to build complementary spring phenology models and compare them to those constructed from historical data alone. Historical trends of phenology are then estimated using these models.

## Materials and Methods

**Figure 1:**
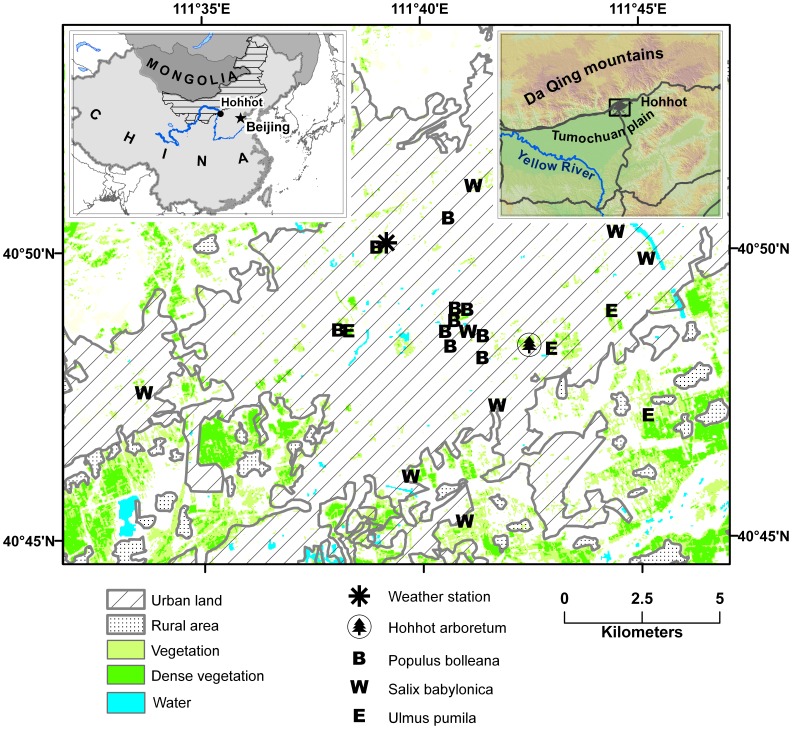
Map of study area showing phenology observations sites. The rectangular area in the upper right inset elevation map with major highways corresponds to the study area extent shown on the main map. Vegetation information is derived from the Normalized Difference Vegetation Index (NDVI) computed from July 28, 2010 Landsat TM image (Vegetation: NDVI = 0.5–0.7, Dense vegetation: NDVI >0.7).

Our study area comprises the capital of Inner Mongolia province of China, Hohhot (40°49' N, 111°41' E), which occupies 2158 km^2^ and has a population of 2 million people. Hohhot is located in cold semi-arid climate characterized by mean annual temperature of 6.6°C and mean annual precipitation of 394 mm and lies at an average elevation of 1050 m above sea level (Fig. 1). The whole area is in the typical steppe zone with natural vegetation dominated by C4 grasses with semi-shrubs and woodland and shrub communities on mountain slopes.

### Historical Monitoring and Contemporaneous Spatial Phenology Data Collection

**Figure 2:**
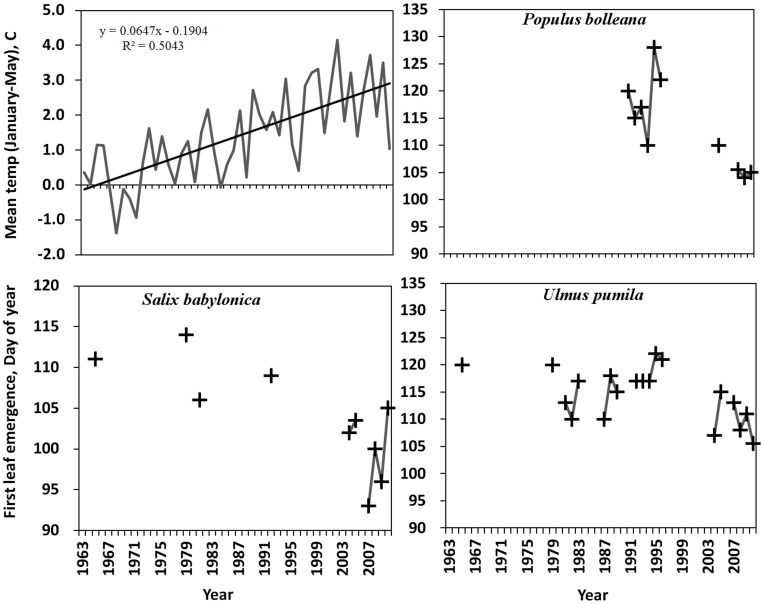
Historical changes in January–May mean air temperature (shown here with the fitted trend line) and timing of first leaf emergence for 3 tree species monitored in Hohhot arboretum during 1963–2010. Lines in phenology graphs connect contiguous data points and illustrate significant data gaps in the time-series.

Phenological data in China have been collected by two major phenology monitoring programs – the Chinese Academy of Science (CAS) network and the Chinese Meteorological Administration (CMA) network [33]. We used data collected under the auspices of CAS in Hohhot arboretum during 1963–1965, 1979–1996, and from 2003 onward. Recorded phenophases include 17 development stages [33]. For this study we focused on several spring phenophases including leaf budburst, first leaf emergence, all leaves unfolded, first bloom, and peak flowering. Three deciduous trees were selected from the multiple woody species monitored in the arboretum: Bolle’s poplar (*Populus bolleana*), Siberian elm (*Ulmus pumila L.*), and weeping (or Peking) willow (*Salix babylonica L.*). These ornamental trees are commonly found in city parks, along street sides, and in the outskirts of the metropolitan area. Although the observation period spans almost five decades, there are significant gaps in data with the total of observation records ranging from 10 to 24 (Fig. 2).

Our field campaign was designed to collect phenological and temperature data during two growing seasons (2010–2011). Considering that potential differences in temperature are influenced by land use, we selected trees in urban parks, urban dense forests (city botanical gardens, plant nurseries), edges of busy streets and major roads, and agricultural hedgerows. No specific permits were required for conducting our field research because all sites were in public areas and did not have endangered or protected species. Three representative healthy individuals of each species growing in close proximity were sampled and tagged at each site. Sites were visited 2–3 times a week during the early to late spring and about 1–2 times a week in the early summer. Julian days of occurrence of phenophases were recorded by several observers following a standardized protocol and by taking digital photographs. In final analyses we use phenophase timing averaged between the three individuals of each species.

### Temperature Data Collection

Historical temperature data for Hohhot were obtained from the China Meteorological Data Sharing Service System of the World Data Center (WDC) using online data retrieval tools (http://cdc.cma.gov.cn/). To collect data at different sites across the city we used 1-Wire iButton® devices including 36 Thermochron® DS-1921G (±1°C accuracy and 0.5°C precision) and 8 Hydrochron® DS-1923 (±0.5°C accuracy and 0.0625°C precision) data loggers. Accuracies of all devices were cross-checked prior to deployment by calibrating in the common room environment to the scientific grade Dynamet (temperature accuracy ±0.25°C) weather station (Dynamax Inc.). Data loggers were deployed in March before maximum daily temperatures reached 0°C. We mounted them at 2 m height on one centrally located tree (one iButton per site) by drilling cavities in tree trunks, spraying paint to protect cavity walls, and inserting PVC pipes (3-cm diameter and 4–5 cm in length). Cavities were facing north to minimize direct solar illumination. Data loggers were placed inside these pipes and cavities shielded by two layers of plastic mesh to allow for ventilation and camouflage from potential vandals and birds. To deal with natural defenses of trees against the wounds we inspected trees during regular visits and removed, if necessary, new cambium growth on outer edges of the cavities. This turned out to be necessary for some of the trees only once in June. Temperature data were recorded at 3-hour interval and converted to daily maximum, minimum, and mean temperature for further analyses. To check whether historical data from Hohhot weather station can be used as a proxy for meteorological conditions in the arboretum, we used nearest (within several hundred meters of each location) spatial sites in similar park-like environments (Fig. 1) and compared their temperature seasonal patterns during 2011. We found remarkably similar temperature patterns with absolute differences predominantly under the accuracy level of our data loggers, which suggests historical temperature data can be justifiably used to model historical phenology.

### Statistical Analyses of Spring Phenology

Phenological models are often based on linear relationships between thermal regime and developmental rates of plants and range from two(three)-parameter ‘spring warming’ regression [34] to more physiologically realistic complex models [35]. There are variants with regards to the form of the explanatory variable included in the ‘spring warming’ model. While some researchers consider mean temperature for periods of different length preceding phenological events [10], [36], others employ the concept of accumulated growing degree-days (AGDD) [35], [37] of the form:
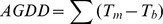
where *T_m_* is the daily mean temperature and *T_b_* is the base temperature (typically 0°C or 5°C). Days with *T_m_*<*T_b_* do not contribute to the sum. The GDD accumulated since the first day of year (DOY) until the DOY at which a specified phenological event occurs signifies a phenophase forcing temperature.

We used a simple ‘spring warming’ AGDD-based linear regression model fitted first to historical data and then to the combined historical and spatial data using the standard ordinary least squares method. In cases where sample size allowed we also constructed regressions from just the spatial data for the purpose of comparison. The model does not take dormancy into account and assumes the chilling requirement necessary for dormancy release has been fulfilled at the start of temperature accumulation. We chose *T_b_* = 5°C as the most commonly used threshold for cereal crops and woody plants of temperate regions [38]. The starting date for GDD accumulation was shortly after March 20 (DOY 80) for both 2010 and 2011. Both the starting date and *T_b_* are essentially identical to those derived by optimization for deciduous forest of the northeastern United States [39]. It is also consistent with some previous observations in Germany suggesting high sensitivity of spring phenology to late spring (March-May) temperatures [39]. We tested the idea that timing of phenophases may be influenced more strongly by AGDD either slightly before or after the average date of phenophase occurrences. This was done by plotting correlations between DOYs of each phenophase and AGDDs with a daily time step and selecting the nearest optimum DOY at which correlation is the highest. Further details on this approach are given in [40], [41].

To assess whether combined spatial and historical phenology data improve predictions based on historical data alone we used combined dataset with the added indicator variable specifying each group (“s” = spatial and “t” = temporal) and the Analysis of Covariance (ANCOVA) [42], [43]. ANCOVA has the general form
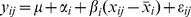
where *y_ij_* is the value of phenophase timing for the *_j_*th observation in the *_i_*th group (spatial vs temporal); *µ* is the overall mean (constant) of phenophase timing; *α_i_* is effect of *_i_*th group on phenophase timing, defined as the difference between each of the group mean and the overall mean; *β_i_* is the slope term for group *i*; *x_ij_* is the value of AGDD for the *_j_*th observation from the *_i_*th group; *X_i_* is the mean value of AGDD for group *i*; and ε*_ij_* is the random error [43]. If the regression slope is the same for both groups (*β_C_*), the model is formulated as



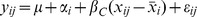



Finally, if there is no interaction and no group effect, the model reduces to a linear regression
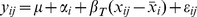
where groups are ignored and slope *β_T_* is the fit to all the data.

**Figure 3:**
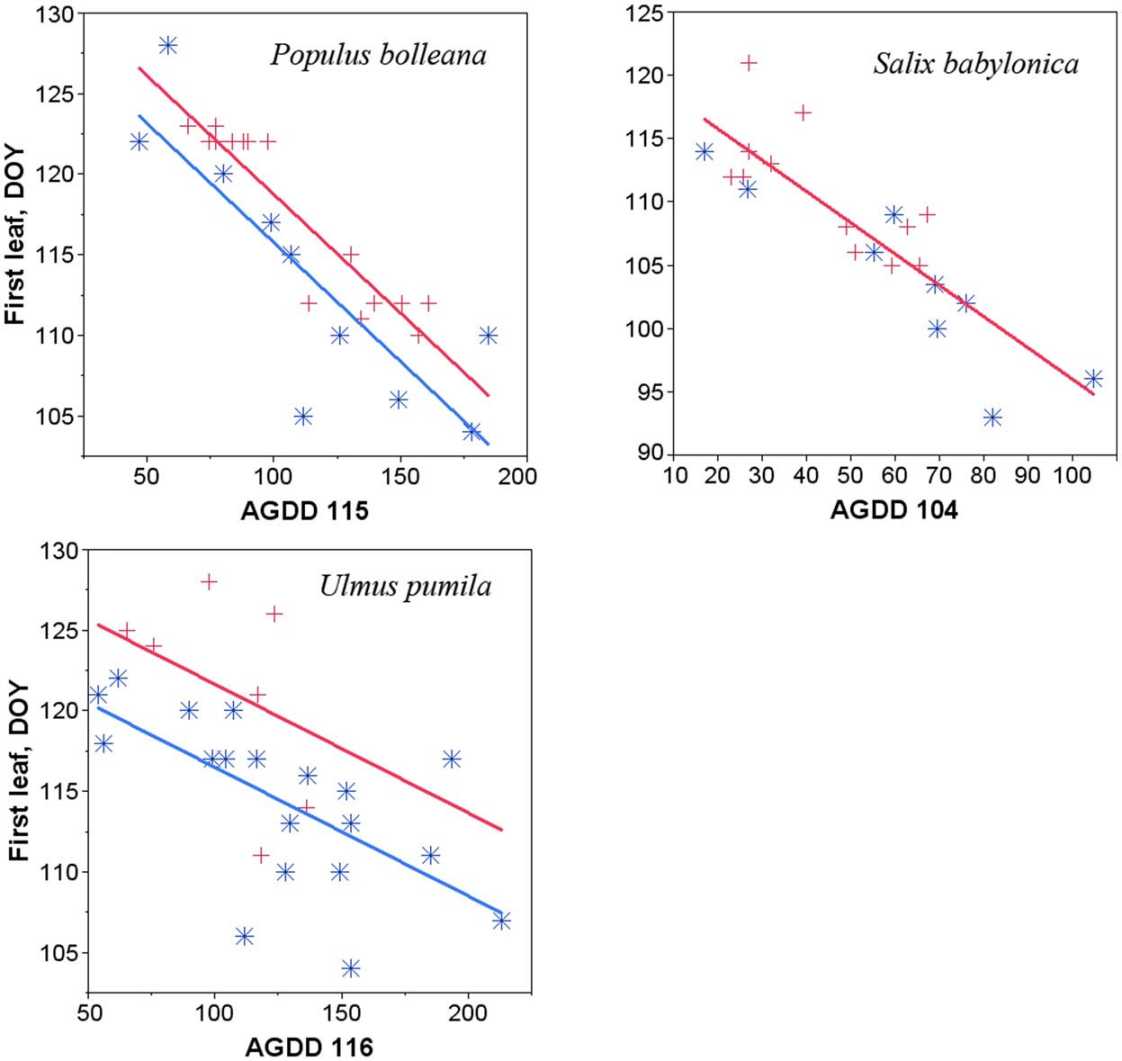
Graphical representation of ANCOVA analysis for the leaf emergence phenophase. Historical data are depicted by blue star symbols and spatial data by red crosses. Linear fits (either a single or two parallel lines) are selected based on ANCOVA. X axis is the growing degree days accumulated to a specified day of year.

By fitting the three models sequentially and using corresponding null hypotheses and the F statistic, we checked whether individual regression lines for the two groups are 1) not parallel (significance of the interaction effect), 2) parallel (significance of the indicator variable), or 3) coincident (single fitted line for the two groups) (Fig. 3). We assessed normality of all variables with Shapiro-Wilk tests. Linearity assumption was checked by fitting a regression with slope = 0 to the residuals. We examined plots of predicted values and residuals to inspect for homoscedasticity. The Durbin-Watson test was used to check for autocorrelation in the residuals.

After selecting best models from combined data (the SFT approach) we applied them to temperature records (1963–2009) and estimated historical gaps in phenology observations. We used these gap-filled time-series to estimate linear trends and compared them to those fitted to historical data alone. This was done, again, by combining these two datasets and fitting separate linear regressions to whole time series and using the same ANCOVA approach for comparing regression slopes. The independent variable here is the year of observation. In addition, we assessed the effect of historical data sample size on these regressions by eliminating one data point at a time, starting from the earliest, and fitting corresponding models. Regression assumptions were checked as described above. All statistical analyses were performed in SAS JMP 7.0.2 software.

## Results

### Temporal vs Spatial Variation of Temperature and Phenology Data

**Figure 4:**
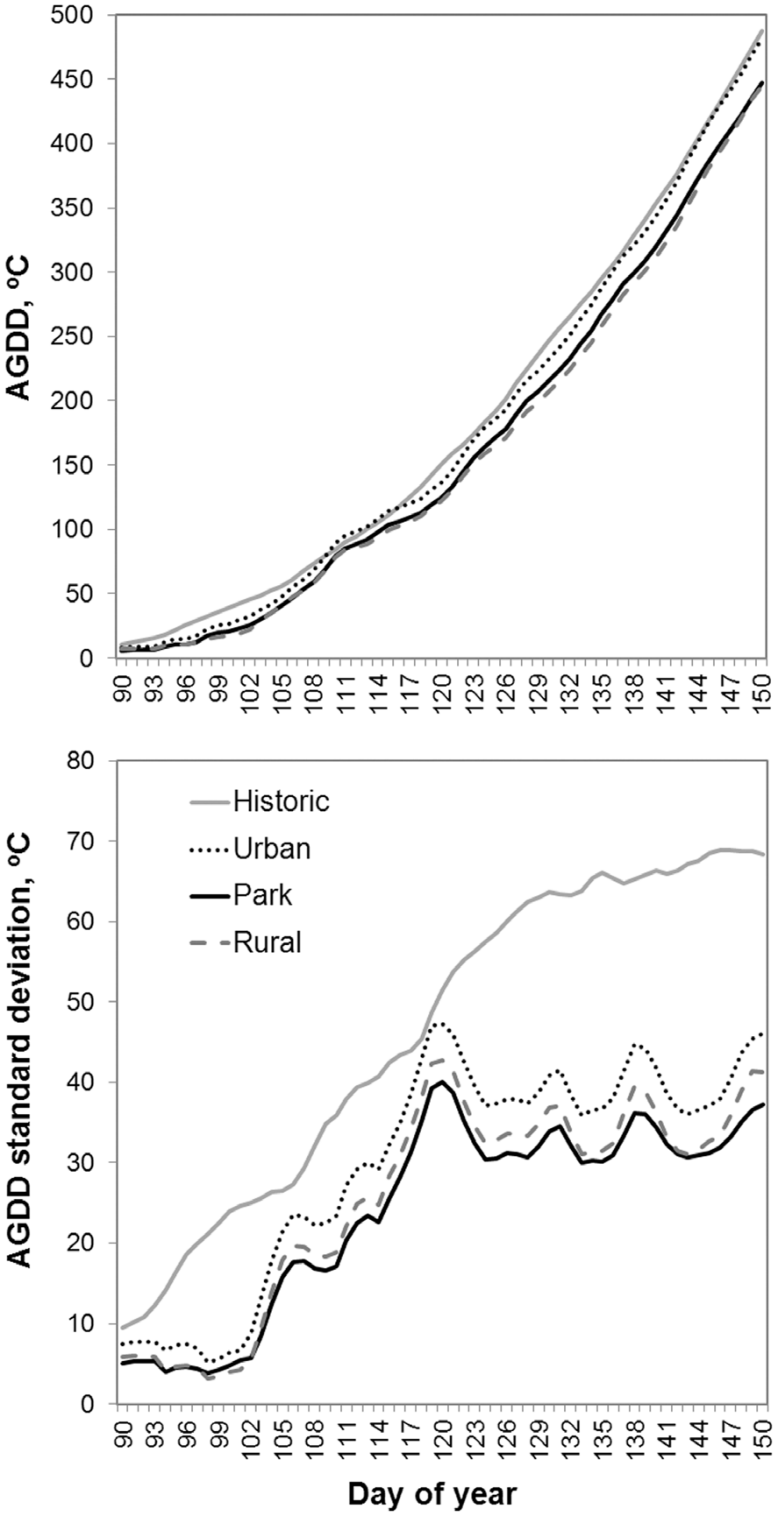
Comparison of mean AGDD (∑>5) accumulation (upper panel) and the associated standard deviation (lower panel) for the period of March,31– May,30. Historic data is the average of years for which phenology observations are available during 1963–2010, and spatial temperature data is the average of two years (2010–11) shown for three major land uses separately.

Spring temperature in Hohhot exhibited a warming trend over the last five decades, although patterns of change in phenology may not be easy to detect (Fig. 2). Note that the spring in 2010 (last point on the graph) was abnormally cooler than the previous 15 years while in 2011 (not shown) it was as warm as in most recent years. To assess whether variability of spatial data exceed or at the level of historical data variability we compared phenology (Table 1) and AGDD (Fig. 4) data statistics. Mean historic AGDD stays close to the mean of urban sites and higher than average AGDD in park and rural locations. However, historic standard deviation is consistently higher (Fig. 4). Both temporal and spatial variation increases with the season progression, although spatial variation increases slower, especially after DOY 120 (Fig. 4). Temporal variation of phenophase timing of all species is also consistently higher than the spatial variation (Table 1). Moreover, this pattern does not always correlate with sample size, e.g. STD of *P. bolleana* is lower despite larger N (Table 1). The comparison of statistical distributions of temperature and phenology suggests our spatial data should probably serve as auxiliary to fill some of the gaps in historical data but cannot be used to completely substitute historical records because spatial variation is consistently lower for both variables.

**Table 1:** Phenology basic statistics.

		Historical (1963–2010)						Spatial (2010–2011)					
Tree species	Stats	BL	L1	LA	BF	F1	PF	BL	L1	LA	BF	F1	PF
*P. bolleana*	Mean	107	114	120	–	–	–	115	117	125	–	–	–
	Min	91	104	112	–	–	–	109	110	120	–	–	–
	Max	125	128	136	–	–	–	122	123	130	–	–	–
	STD	10.2	8.0	7.7	–	–	–	5.4	5.4	2.5	–	–	–
	N	10	10	9	–	–	–	17	17	17	–	–	–
*S. babylonica*	Mean	94	102	113	100	108	112	109	111	124	114	118	122
	Min	88	93	102	88	97	105	103	105	118	108	117	117
	Max	110	114	132	113	117	131	120	121	131	119	119	126
	STD	7.6	6.9	8.6	10.8	6.8	8.4	4.4	4.6	4.3	5.5	1.2	4.2
	N	9	9	11	7	9	8	14	14	14	3	3	5
*U. pumila*	Mean	102	114	122	–	99	109	120	122	136	100	104	108
	Min	76	104	110	–	84	88	109	111	126	93	97	101
	Max	123	122	130	–	119	127	124	128	144	105	113	115
	STD	12.9	5.2	5.1	–	9.3	12.2	6.0	6.1	5.9	6.6	6.2	5.0
	N	16	19	21	–	17	22	5	8	8	5	6	5

Notes: BL = leaf budburst, L1 = first leaf fully out, LA = all leaves on plant are unfolded, BF = flower visible, F1 = first bloom, PF = peak flowering. STD = standard deviation and N = number of years/sites.

### Differences in Regression Slopes of Combined Models

There is no difference in the way the timing of phenophases from historical data and that from spatial data change with increasing AGDD (Table 2, note large *p*-values for the interaction term effect). Small *p*-values for the indicator variable effect and *t*-test results of the least squares means (not shown here) suggest two parallel lines can be fit to the combined dataset for about half of the phenophases. This information was used to fit regressions using combined historical and spatial data. Those phenophases with high *p*-values for the indicator variable effect were fitted using the parallel lines model, while others were fitted using the single line model. We then evaluated outputs from these regressions with those constructed using just the historical data by considering statistical significance and standard error (SE) of slopes and percent of phenophase timing variance explained by these models (Table 3). Most slopes and SE are similar when compared between the two groups of models (historical vs combined). We note some improvements, mainly because of considerable increases of R^2^_adj_. Combining temporal and spatial data for *P. bolleana* increased the degrees of freedom and improved R^2^_adj_ of leaf budburst and first leaf appearance phenophases (Table 3). Similar improvements are evident when leaf budburst and flowering phenology of *Salix babylonica* are examined. Historical correlations of all *Ulmus pumila* phenophases with AGDD were quite low. Combining historical with spatial observations increased R^2^_adj_ of leaf phenophases to some degree but the slope of leaf budburst remained non-significant (Table 3).

**Table 2:** Analysis of covariance (ANCOVA) testing differences between linear regression fits to historical and spatial phenological observations.

		Non-parallel lines (interaction		Parallel lines (indicator variable effect)		Co-incident lines	
Tree species	Phenophase	F	*p*	F	*p*	F	*p*
*P. bolleana*	BL	0.12_1,23_	0.73	6.20_1,24_	**0.02**	77.34_1,25_	<**.001**
	L1	0.06_1,23_	0.81	5.76_1,24_	**0.02**	70.65_1,25_	<**.001**
	LA	2.38_1,22_	0.13	0.64_1,23_	0.43	33.24_1,24_	**0.01**
*S. babylonica*	BL	0.002_1,18_	0.97	23.81_1,19_	<**.001**	41.09_1,20_	<**.001**
	L1	0.08_1,18_	0.78	3.96_1,19_	0.06	55.92_1,20_	<**.001**
	LA	0.08_1,20_	0.78	7.93_1,21_	**0.01**	25.52_1,22_	<**.001**
	BF	0.16_1,6_	0.70	0.26_1,7_	0.63	10.74_1,8_	**0.01**
	F1	0.05_1,8_	0.83	1.02_1,9_	0.34	21.56_1,10_	<**.001**
	PF	0.02_1,8_	0.89	0.05_1,9_	0.83	18.27_1,10_	**0.02**
*U. pumila*	BL	0.21_1,16_	0.66	4.73_1,17_	**0.04**	3.36_1,18_	0.08
	L1	0.64_1,22_	0.43	6.01_1,23_	**0.02**	14.38_1,24_	<**.001**
	LA	0.01_1,23_	0.91	25.26_1,24_	<**.001**	8.29_1,25_	**0.01**
	F1	0.06_1,18_	0.81	0.001_1,19_	0.98	10.13_1,20_	**<.001**

Notes: Phenophases are same as in Table 1. Numerator and denominator degrees of freedom are listed as subscripts for each F-value. Bold face font indicates significance at *p*<0.05.

**Table 3:** Comparison of best phenological regression models for combined and historical data.

		Historical data					Combined data				
Tree species	Phenophase	Slope	SE	t*_df_*	*p*	R^2^_adj_	Slope	SE	t*_df_*	*p*	R^2^_adj_
*P. bolleana*	BL*	−0.23	0.05	−4.31_8_	<**.001**	0.66	−0.22	0.03	−8.60_24_	<**.001**	0.79
	L1*	−0.14	0.03	−4.25_8_	<**.001**	0.65	−0.15	0.02	−9.14_24_	<**.001**	0.77
	LA	−0.22	0.06	−3.57_7_	**<.001**	0.59	−0.22	0.04	−5.77_24_	**<.001**	0.56
*S. babylonica*	BL*	−0.24	0.05	−4.54_7_	<**.001**	0.71	−0.24	0.04	−5.72_19_	<**.001**	0.84
	L1	−0.23	0.04	−5.42_7_	<**.001**	0.78	−0.25	0.03	−7.48_20_	<**.001**	0.72
	LA	−0.14	0.02	−5.84_9_	<**.001**	0.77	−0.08	0.02	−3.54_21_	**0.002**	0.61
	BF*	−0.22	0.13	−1.65_5_	0.16	0.22	−0.27	0.08	−3.28_8_	**0.01**	0.52
	F1*	−0.17	0.06	−2.87_7_	<**.001**	0.47	−0.20	0.04	−4.64_10_	**0.001**	0.65
	PF*	−0.13	0.05	−2.64_6_	**0.04**	0.46	−0.12	0.03	−4.27_10_	**0.002**	0.61
*U. pumila*	BL*	−0.09	0.08	−1.11_14_	0.29	0.02	−0.10	0.07	−1.37_17_	0.19	0.26
	L1*	−0.07	0.02	−3.31_17_	<**.001**	0.36	−0.08	0.02	−3.47_23_	**0.002**	0.46
	LA*	−0.04	0.02	−2.36_19_	**0.03**	0.19	−0.04	0.02	−2.30_24_	**0.03**	0.60
	F1	−0.10	0.04	−2.76_15_	**0.015**	0.29	−0.10	0.03	−3.18_20_	**0.005**	0.30

Notes: Phenophases are same as in Table 1. SE = standard error of the slope. Bold face font indicates significance at *p*<0.05.

* denotes phenophases for which combining historical and spatial data resulted in improvements.

### Analysis of Temporal Trends of Phenology

**Figure 5:**
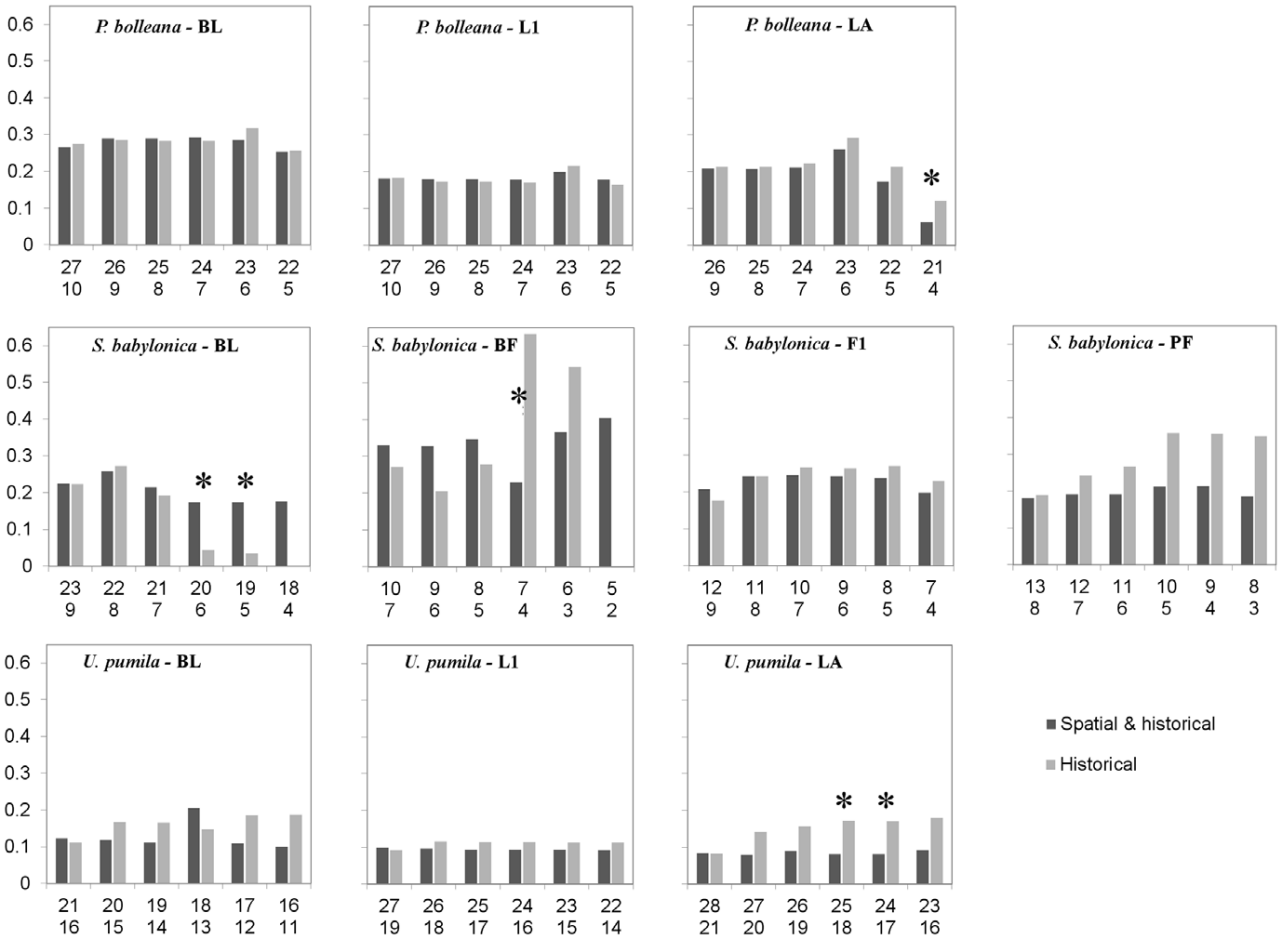
Absolute slopes (Y-axis) of linear fits to phenological time-series (1963–2009) predicted based on phenology regression models from combined data and those constructed from historical records. All slopes are negative and significant at p<0.05. Numbers along the X-axis are sample sizes (top = combined and bottom = historical) used to develop phenology models. They decrease according to the elimination of one historical data point at a time, starting from the earliest. * indicate significance (p<0.05) of slope difference based on ANCOVA analysis. Phenophases are same as in Table 1.

Comparing between linear fits to historical data and to the full time-series of predictions from best combined (SFT) models suggests no difference in slopes, although the former are often not significant due to small data sizes (Table 4). Phenology observations of *P. bolleana* started only in 1991, so large slope differences are mostly the result of temporal mismatch between the two datasets compared (Fig. 2). This confirms high dependence of trend estimation on such factors as starting year, end year, and duration of analysis [14]. Furthermore, linear trends of time-series predicted by historical data alone and those by best combined models are also similar in their rate of change (Fig. 5, first pair of bars in each graph) suggesting historical data are actually sufficient for model building and trend estimations. All slopes are negative and translate into phenophase advancement of 2–3 days for *P. bolleana* and *S. babylonica* and one day per decade for *U. pumila*. The advantage of combining spatial and temporal data becomes evident when historical data size is further reduced (Fig. 5). Resulting slopes are shown for historical data reduced by half or more (and the corresponding reduction of combined data), except for *U. pumila* whose full sequence is not shown here. Most slopes of linear trends fitted to predictions from combined data remain at the same level, but they are less stable for the LA phenophase of *P. bolleana* and BF phenophase of *S. babylonica*. Slopes of linear trends fitted to predictions from historical data are more sensitive to data size reduction, especially for BL, BF, and PF phenophases of *S. babylonica* and LA phenophase of *U. pumila*. Several statistical differences between slopes for the two species (Fig. 5) are the result of this instability and support the idea that combining spatial and temporal data is beneficial, especially when the number of historical records is really small, e.g. 6 or 4. Interestingly, the significant difference in slopes for LA of *P. bolleana* is caused by slope instability for the combined dataset.

**Table 4:** Comparison of regression fits to historical data and to the time-series of predictions (1963–2009) from phenology models developed based on combined data.

		Linear fit to historical data alone				Linear fit to predictions from combined data			
Tree species	Phenophase	Slope	SE	t*_df_*	*p*	Slope	SE	t*_df_*	*p*
*P. bolleana*	BL*	−0.96	0.35	−2.77_8_	**0.02**	−0.29	0.06	−5.72_45_	<**.001**
	L1*	−0.79	0.29	−2.72_7_	**0.02**	−0.21	0.06	−3.66_45_	**<.001**
*S. babylonica*	BL	−0.37	0.11	−3.70_7_	**0.008**	−0.25	0.05	−4.72_45_	<**.001**
	BF	−0.52	0.36	−1.42_5_	0.21	−0.38	0.10	−3.71_45_	<**.001**
	F1	−0.34	0.20	−1.74_6_	0.13	−0.24	0.05	−3.71_45_	<**.001**
	PF	−0.31	0.09	−3.62_5_	**0.015**	−0.22	0.05	−4.28_45_	**<.001**
*U. pumila*	BL	−0.10	0.23	−0.44_13_	0.67	−0.14	0.03	−4.08_45_	<**.001**
	L1	−0.19	0.10	−1.83_16_	0.09	−0.11	0.03	−3.64_45_	<**.001**
	LA	−0.12	0.09	−1.24_18_	0.23	−0.10	0.02	−4.92_45_	**<.001**

Notes: Phenophases are same as in Table 1. Bold face font indicates significance at *p*<0.05.

* denotes phenophases for which slope difference is statistically significant (*p*<0.05) according to ANCOVA analysis.

## Discussion

### Observed Improvements in Phenology Models and Estimation of Historical Trends Achieved by SFT Substitution

SFT substitution appears to be expedient for phenology modeling of large woody plants for which overall historical sample sizes are either small or the continuity of time-series is interrupted by substantial gaps. This is supported by our findings for *P. bolleana* and *S. babylonica* that each had only 8–10 observations for different phenophases. By collecting additional data we were able to fill gaps in the lower AGDD and later phenophase timing space of scatter plots for these variables, as well as adding more points to the middle (Fig. 3). It eventually improved phenology models but did not actually affect estimates of historical rates of change in phenophase timing. As expected, significant differences in slopes of trend lines were detected only for the reduced dataset of *S. babylonica*, though *P. bolleana* did not fit this pattern. The results illustrate the importance of data availability for just one or two earliest years for prediction of realistic linear trends. Our findings for the other species are variable. Two other species of poplar were not as common in the urban landscape, so adding scanty spatial data did not affect regression models constructed with historical data. Although *U. pumila* had one of the most complete historical records, we could not identify strong enough relationships of phenophase timing with temperature. Combining it with spatial data provided only a small improvement.

The highest percent variance was consistently explained by AGDD in either BL or F1 models. We attribute this to the more accurate and unambiguous detection of these phenophases by different observers, while human errors are presumably higher for the LA phenophase. During our field campaign flowering phenology was only observed in *S. babylonica* and *U. pumila*. Combining these observations with temporal data improved phenology models sufficiently, but percent of variance explained by temperature remained low or relatively moderate.

Chen and Xu [40] recently demonstrated high contrasts in historical trends of growing season onset of *U. pumila* across the entire temporal zone of China. In particular, among the four stations centered on our study area, two showed significant advancing trends for the beginning of growing season (4–7 days/decade), while one station showed a delayed trend of 3 days/decade, and another exhibited almost no change (stations 17–20 in Table 1 of Chen and Xu (2011)). However, with one exception, none of those trends were statistically significant, which is also demonstrated by our results for this tree (Table 4). As noted earlier, these trends are weak because of high year-to-year variation in phenophase timing and its lower correlation with AGDD. When we substituted historical data gaps with those estimated from our regression models, trend lines became statistically significant, but slopes remained similar.

### Potential for Improving the SFT Substitution in Phenology Research

We believe there are still underexplored opportunities in bringing temporal and spatial perspectives together, which deserve further research efforts in phenology studies. Most our spatial data did not cover whole ranges of historical data so it could not be used to predict historical dynamics per se, but it was useful as complementary data. We envision most urban temperature/phenology gradients surveyed within a single year cannot surpass variability of historical records. Combining data from two or more years, as well as incorporating sharp altitudinal gradients [44], where possible, is expected to alleviate this shortcoming. Collecting spatial phenological data is labor intensive and may sometimes be thwarted by logistical challenges. Recent technological advances can alleviate some of these problems. With the increase in remote sensing capabilities, introduction of advanced monitoring devices, and development of cyber-infrastructure [45], high quality spatial data on phenology can be collected.

**Figure 6:**
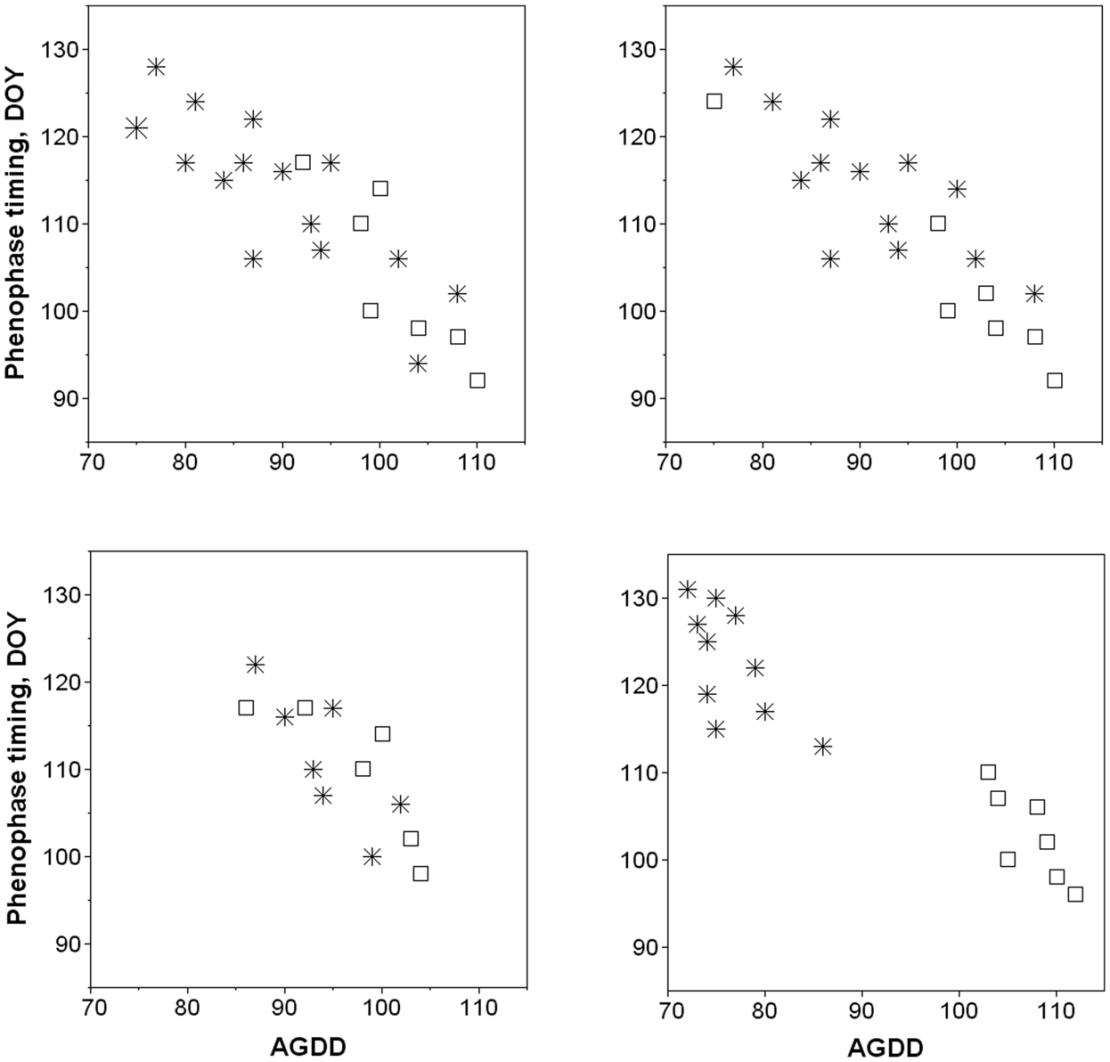
Scatterplots illustrating hypothetical situations when spatial data (star symbols) are combined with historical data (squares).

In situations when historical data are too few to construct robust statistical models and make inferences, the sheer benefit of the SFT approach is in boosting the sample size and gaining statistical power. This was partly demonstrated by our analyses although we did not have independent datasets to conduct true validation, which is one of the major shortcomings of the current study. We also believe the success of the SFT substitution depends much on both temporal and spatial data structures and illustrate this in the hypothetical examples (Fig. 6). The two upper graphs show favorable situations when adding spatial data sufficiently improves phenology models. This is especially true for the upper right case where both the total number of data points is substantially increased and the range of spatial data far exceeded the historical range. Two lower graphs are examples of unfavorable situations when adding spatial data is unlikely to help in producing useful models, especially in the lower right case. In addition, if long-term observations are terminated because of land use changes or the loss of trees at a site, the SFT may serve as an actual physical substitute for the discontinued time-series.

Since a linear relationship between temperature and phenology is often assumed, it can be theoretically predicted with a limited number of points. However, if the true relationship is non-linear, a sufficient number of observations are required to build a reasonable model. By the same token, historical trends of phenology do not need to be linear. For example, one may be interested in analyzing historical change by fitting a locally weighted scatterplot smoothing (LOESS) regression, which requires a large, densely sampled dataset. The SFT substitution has great potential for handling such non-linearity, provided that the combined dataset meets such data density requirements.

Finally, the statistical framework in our study was quite simple, but the SFT substitution for estimation of trends in plant phenology can certainly benefit from more elaborate approaches, which include linear models combined with Gaussian Mixture Models [46], Bayesian statistics [13], penalized regression [47], or Generalised Additive Models for Location, Scale and Shape (GAMLSS) [48].

### Theoretical Assumptions and Practical Limitations

SFT substitution implementation in phenology research is faced with a number of assumptions and uncertainties. The most underlying assumption is that plants respond to spatial patterns of temperature in the same way they respond to year-to-year temperature variation. Besides, climate-based phenology models rely on the simplifying assumption of both temporal and spatial stationarity, i.e. phenology responses to environmental cues are stable through time and across space [49]. The validity of these assumptions remains to be tested. Another important limitation of our study is likely correlated error structure in repeated phenological observations from multiple locations [50], which may increase the likelihood of type I error in hypothesis testing and create problems in statistical inference. Drawing upon the discussion of models with missing data in repeated (longitudinal) observations Kelly [50] suggested several statistical methods to deal with the issue, which should be considered in future research.

In our study we did not consider other environmental cues - photoperiod, chilling requirement, or precipitation. The effect of photoperiod is only relevant when studies are conducted at broad scales, or if they involve late successional plant species known to be under photoperiodic control [51]. Besides, photoperiod does not vary from one year to another, and it is unlikely to influence inter-annual variation of phenology in a single place [52]. Precipitation was not considered in our study, partly because of our inability to measure its spatial variation across the urban area and partly because of widespread irrigation. For simplicity and for the ease of comparison between spatial and temporal data we assumed all these cues are not as critical as forcing temperature, a primary trigger of spring phenology in temperate regions [6], [53]–[55]. The relative importance of environmental factors in triggering phenophases, exact molecular and physiological mechanisms of phenology, and the relative role of genetic differences vs plastic responses to environmental heterogeneity are still open-ended questions for these and most other plant species [56]. Weaker correlations of phenophase timing with thermal regime found for some trees in our study are likely because of the omission of some these factors.

Although we selected mature trees, we did not control for internal factors of phenology - tree age and genotype. Species identity in our study was checked without examining their genetic structures. This is another important limitation, which we plan to address directly in future research. Previous analysis using common garden experiments [57] suggested temporal deciduous trees exhibit high phenotypic plasticity, i.e. effects of locational differences may override the effect of genetic differences of species.

Major uncertainties in phenology modeling are related to the fact that phenological data are derived from visual observations, not from instrumental measurements [46]. Observations are prone to bias and misinterpretation of protocols, which increase with the increase of the number of observers. Finally, one particular difficulty of studying urban phenology is rapid land use changes and unexpected management decisions. These processes change plant distribution patterns and microclimate of each site, which may further affect the reliability of long-term studies. An additional challenge is related to safeguarding meteorological and photographic instrumentation in places of high population density.

### Conclusions

In our study, we proposed and tested the usefulness of the indirect SFT substitution whereby quality spatial phenology and temperature data obtained in the same urban landscape are used as a leverage to fill gaps in scarce historical records and build complementary phenology models. Because of the legacy of phenological monitoring in or near populated places urban regions are well suited for applying the SFT substitution and should receive more attention from researchers developing approaches to examining phenology and climatic trends. Not only they create temperature gradients (UHI), but also modify other factors that may affect phenology, including CO_2_ enrichment, environmental pollution, reduced pollination by insects, and, potentially, light pollution.

Potential benefits in applying the SFT substitution in phenology studies are summarized as follows. The approach allows increasing the overall sample size and developing more accurate statistical models of phenology when historic data are scarce. Important data gaps in phenological records can be filled in, provided that a strong relationship of phenophase timing with environmental triggers (temperature) is established. Slopes of historical linear trends may be estimated more accurately. We conclude there is considerable potential for exploring SFT substitution analyses in phenology studies, especially those conducted in urban settings.

## References

[1] Badeck F-W, Bondeau A, Böttcher K, Doktor D, Lucht W, et al (2004) Responses of spring phenology to climate change. New Phytologist 162: 295–309.

[2] Morin X, Roy J, Sonié L, Chuine I (2010) Changes in leaf phenology of three European oak species in response to experimental climate change. New Phytologist 186: 900–910. 20406403 10.1111/j.1469-8137.2010.03252.x

[3] Schwartz MD, Ahas R, Aasa A (2006) Onset of spring starting earlier across the Northern Hemisphere. Global Change Biology 12: 343–351.

[4] Menzel A (2003) Plant Phenological “Fingerprints”: Detection of Climate Change Impacts. In: Schwartz MD, editor. Phenology: An Integrative Environmental Science. Dordrecht/Boston/London: Kluwer. 319–330.

[5] Chmielewski F-M, Rötzer T (2001) Response of tree phenology to climate change across Europe. Agricultural and Forest Meteorology 108: 101–112.

[6] Schwartz MD, editor (2003) Phenology: An Integrative Environmental Science. Dordrecht/Boston/London: Kluwer.

[7] Miller-Rushing AJ, Primack RB (2008) Global warming and flowering times in Thoreau’s Concord: A community perspective. Ecology 89: 332–341. 18409423 10.1890/07-0068.1

[8] Primack RB, Ibáñez I, Higuchi H, Lee SD, Miller-Rushing AJ, et al (2009) Spatial and interspecific variability in phenological responses to warming temperatures. Biological Conservation 142: 2569–2577.

[9] Menzel A, Sparks TH, Estrella N, Koch E, Aasa A, et al (2006) European phenological response to climate change matches the warming pattern. Global Change Biology 12: 1969–1976.

[10] Bertin RI (2008) Plant Phenology And Distribution In Relation To Recent Climate Change. The Journal of the Torrey Botanical Society 135: 126–146.

[11] Parmesan C (2006) Ecological and Evolutionary Responses to Recent Climate Change. Annual Review of Ecology, Evolution, and Systematics 37: 637–669.

[12] Heide OM (2003) High autumn temperature delays spring bud burst in boreal trees, counterbalancing the effect of climatic warming. Tree Physiology 23: 931–936. 14532017 10.1093/treephys/23.13.931

[13] Schleip C, Menzel A, Dose V (2010) Bayesian Methods in Phenology. In: Hudson IL, Keatley MR, editors. Phenological Research: Methods for Environmental and Climate Change Analysis. Dordrecht/Heidelberg/London/New York: Springer. 229–254.

[14] Sparks TH, Tryjanowski P (2005) The detection of climate impacts: some methodological considerations. International Journal of Climatology 25: 271–277.

[15] Schaber J, Badeck F-W (2002) Evaluation of methods for the combination of phenological time series and outlier detection. Tree Physiology 22: 973–982. 12359524 10.1093/treephys/22.14.973

[16] Linkosalo T, Häkkinen R, Hari P (1996) Improving the reliability of a combined phenological time series by analyzing observation quality. Tree Physiology 16: 661–664. 14871705 10.1093/treephys/16.7.661

[17] Häkkinen R, Linkosalo T, Hari P (1995) Methods for combining phenological time series: application to bud burst in birch (Betula pendula) in Central Finland for the period 1896–1955. Tree Physiology 15: 721–726. 14965990 10.1093/treephys/15.11.721

[18] Miller-Rushing AJ, Primack RB, Primack D, Mukunda S (2006) Photographs and herbarium specimens as tools to document phenological changes in response to global warming. American Journal of Botany 93: 1667–1674. 21642112 10.3732/ajb.93.11.1667

[19] Neil K, Landrum L, Wu J (2010) Effects of urbanization on flowering phenology in the metropolitan Phoenix region of USA: Findings from herbarium records. Journal of Arid Environments 74: 440–444.

[20] Little RJA, Rubin DR (1987) Statistical Analysis with Missing Data. New York, USA: John Wiley & Sons.

[21] Dempster AP, Laird NM, Rubin DB (1977) Maximum Likelihood from Incomplete Data via the EM Algorithm. Journal of the Royal Statistical Society Series B (Methodological) 39: 1–38.

[22] Post ES, Pedersen C, Wilmers CC, Forchhammer MC (2008) Phenological sequences reveal aggregate life history response to climate warming. Ecology 89: 363–370. 18409426 10.1890/06-2138.1

[23] Chapin FS, Shaver GR, Giblin AE, Nadelhoffer KJ, Laundre JA (1995) Responses of Arctic Tundra to Experimental and Observed Changes in Climate. Ecology 76: 694–711.

[24] Vitousek PM, Matson PA (1991) Gradient Analysis of Ecosystems. In: Cole J, Lovett GM, Findlay S, editors. Comparative analyses of ecosystems: patterns, mechanisms, and theories. New York: Springer-Verlag. 287–298.

[25] Wolkovich EM, Cook BI, Allen JM, Crimmins TM, Betancourt JL, et al (2012) Warming experiments underpredict plant phenological responses to climate change. Nature 485: 494–497. 22622576 10.1038/nature11014

[26] Dunne JA, Saleska SR, Fischer ML, Harte J (2004) Integration experimental and gradient methods in ecological climate change research. Ecology 85: 904–916.

[27] Ziska LH, Gebhard DE, Frenz DA, Faulkner S, Singer BD, et al (2003) Cities as harbingers of climate change: Common ragweed, urbanization, and public health. Journal of Allergy and Clinical Immunology 111: 290–295. 12589347 10.1067/mai.2003.53

[28] Shustack D, Rodewald A, Waite T (2009) Springtime in the city: exotic shrubs promote earlier greenup in urban forests. Biological Invasions 11: 1357–1371.

[29] Mimet A, Pellissier V, Quénol H, Aguejdad R, Dubreuil V, et al (2009) Urbanisation induces early flowering: evidence from *Platanus acerifolia* and *Prunus cerasus* . International Journal of Biometeorology 53: 287–298. 19219464 10.1007/s00484-009-0214-7

[30] Gazal R, White MA, Gillies R, Rodemaker ELI, Sparrow E, et al (2008) GLOBE students, teachers, and scientists demonstrate variable differences between urban and rural leaf phenology. Global Change Biology 14: 1568–1580.

[31] Pickett STA (1989) Space-for-time substitution as an alternative to long-term studies. In: Likens GE, editor. Long-Term Studies in Ecology: Approaches and Alternatives. New York, Berlin: Springer-Verlag. 110–135.

[32] Fukami T, Wardle DA (2005) Long-term ecological dynamics: reciprocal insights from natural and anthropogenic gradients. Proceedings of the Royal Society B-Biological Sciences 272: 2105–2115. 10.1098/rspb.2005.3277 PMC1559953 16191623

[33] Chen X (2003) Phenological Data, Networks, and Resarch: East Asia. In: Schwartz MD, editor. Phenology: An Integrative Environmental Science. Dordrecht/Boston/London: Kluwer. 11–25.

[34] Rötzer T, Grote R, Pretzsch H (2004) The timing of bud burst and its effect on tree growth. International Journal of Biometeorology 48: 109–118. 14564495 10.1007/s00484-003-0191-1

[35] Chuine I, Kramer K, Hanninen H (2003) Plant Development Models. In: Schwartz MD, editor. Phenology: An Integrative Environmental Science. Dordrecht/Boston/London: Kluwer. 11–26.

[36] Chmielewski F-M, Rötzer T (2002) Annual and spatial variability of the beginning of growing season in Europe in relation to air temperature changes. Climate Research 19: 257–264.

[37] Thompson R, Clark R (2006) Spatio-temporal modelling and assessment of within-species phenological variability using thermal time methods. International Journal of Biometeorology 50: 312–322. 16506032 10.1007/s00484-005-0017-4

[38] Wielgolaski FE (1999) Starting dates and basic temperatures in phenological observations of plants. International Journal of Biometeorology 42: 158–168.

[39] Fisher JI, Richardson AD, Mustard JF (2007) Phenology model from surface meteorology does not capture satellite-based greenup estimations. Global Change Biology 13: 707–721.

[40] Chen X, Xu L (2011) Phenological responses of *Ulmus pumila* (Siberian Elm) to climate change in the temperate zone of China. International Journal of Biometeorology 56: 695–706. 21805230 10.1007/s00484-011-0471-0

[41] Matsumoto K, Ohta T, Irasawa M, Nakamura T (2003) Climate change and extension of the Ginkgo biloba L. growing season in Japan. Global Change Biology 9: 1634–1642.

[42] Neter J, Kutner MH, Nachtsheim CJ, Wasserman W (1996) Applied Linear Statistical Models. Boston, Massachusetts: McGraw-Hill/Irwin. 1408 p.

[43] Gotelli NJ, Ellison AM (2004) A Primer of Ecological Statistics. Sunderland, MA USA: Sinauer Associates, Inc.

[44] Jochner S, Sparks T, Estrella N, Menzel A (2011) The influence of altitude and urbanisation on trends and mean dates in phenology (1980–2009). International Journal of Biometeorology: 1–8. 10.1007/s00484-011-0444-3 21604152

[45] Morisette JT, Richardson AD, Knapp AK, Fisher JI, Graham EA, et al (2009) Tracking the rhythm of the seasons in the face of global change: phenological research in the 21st century. Frontiers in Ecology and the Environment 7: 253–260.

[46] Schaber J, Badeck F-W, Doktor D, von Bloh W (2010) Combining Messy Phenological Time Series. In: Hudson IL, Keatley MR, editors. Phenological Research: Methods for Environmental and Climate Change Analysis. Dordrecht/Heidelberg/London/New York: Springer. 147–158.

[47] Roberts A (2008) Exploring relationships between phenological and weather data using smoothing. International Journal of Biometeorology 52: 463–470. 18193297 10.1007/s00484-007-0141-4

[48] Hudson IL, Kim SW, Keatley MR (2010) Climatic Influences on the Flowering Phenology of Four Eucalypts: A GAMLSS Approach. In: Hudson IL, Keatley MR, editors. Phenological Research: Methods for Environmental and Climate Change Analysis. Dordrecht/Heidelberg/London/New York: Springer. 209–228.

[49] Pau S, Wolkovich EM, Cook BI, Davies TJ, Kraft NJB, et al (2011) Predicting phenology by integrating ecology, evolution and climate science. Global Change Biology 17: 3633–3643.

[50] Kelly N (2010) Accounting for Correlated Error Structure Within Phenological Data: a Case Study of Trend Analysis of Snowdrop Flowering. In: Hudson IL, Keatley MR, editors. Phenological Research: Methods for Environmental and Climate Change Analysis. Dordrecht/Heidelberg/London/New York: Springer. 271–298.

[51] Körner C, Basler D (2010) Phenology Under Global Warming. Science 327: 1461–1462. 20299580 10.1126/science.1186473

[52] Chuine I, Cour P, Rousseau DD (1999) Selecting models to predict the timing of flowering of temperate trees: implications for tree phenology modelling. Plant, Cell & Environment 22: 1–13.

[53] Polgar CA, Primack RB (2011) Leaf-out phenology of temperate woody plants: from trees to ecosystems. New Phytologist 191: 926–941. 21762163 10.1111/j.1469-8137.2011.03803.x

[54] Sparks TH, Jeffree EP, Jeffree CE (2000) An examination of the relationship between flowering times and temperature at the national scale using long-term phenological records from the UK. International Journal of Biometeorology 44: 82–87. 10993562 10.1007/s004840000049

[55] Fitter AH, Fitter RSR, Harris ITB, Williamson MH (1995) Relationships Between First Flowering Date and Temperature in the Flora of a Locality in Central England. Functional Ecology 9: 55–60.

[56] Forrest J, Miller-Rushing AJ (2010) Toward a synthetic understanding of the role of phenology in ecology and evolution. Philosophical Transactions of the Royal Society B: Biological Sciences 365: 3101–3112. 10.1098/rstb.2010.0145 PMC2981948 20819806

[57] Kramer K (1995) Phenotypic plasticity of the phenology of seven European tree species in relation to climatic warming. Plant, Cell & Environment 18: 93–104.

